# Directly addressable GaN-based nano-LED arrays: fabrication and electro-optical characterization

**DOI:** 10.1038/s41378-020-00198-y

**Published:** 2020-10-19

**Authors:** Daria D. Bezshlyakh, Hendrik Spende, Thomas Weimann, Peter Hinze, Steffen Bornemann, Jan Gülink, Joan Canals, Joan Daniel Prades, Angel Dieguez, Andreas Waag

**Affiliations:** 1 Institute of Semiconductor Technology, Technische Universität Braunschweig, Hans-Sommer Str. 66, 38106 Braunschweig, Germany; 2 Laboratory for Emerging Nanometrology, Langer Kamp 6 a/b, 38106 Braunschweig, Germany; 3 Epitaxy Competence Center ec2, Technische Universität Braunschweig, Hans-Sommer Str. 66, 38106 Braunschweig, Germany; 4 Nanostructuring and Clean Room Center Infrastructure, Physikalisch-Technische Bundesanstalt, Bundesallee 100, 38116 Braunschweig, Germany; 5 Department of Electronic and Biomedical Engineering, University of Barcelona, Carrer Marti i Franques 1, 08028 Barcelona, Spain

**Keywords:** Nanophotonics and plasmonics, Micro-optics

## Abstract

The rapid development of display technologies has raised interest in arrays of self-emitting, individually controlled light sources atthe microscale. Gallium nitride (GaN) micro-light-emitting diode (LED) technology meets this demand. However, the current technology is not suitable for the fabrication of arrays of submicron light sources that can be controlled individually. Our approach is based on nanoLED arrays that can directly address each array element and a self-pitch with dimensions below the wavelength of light. The design and fabrication processes are explained in detail and possess two geometries: a 6 × 6 array with 400 nm LEDs and a 2 × 32 line array with 200 nm LEDs. These nanoLEDs are developed as core elements of a novel on-chip super-resolution microscope. GaN technology, based on its physical properties, is an ideal platform for such nanoLEDs.

## Tiny lights enable molecular-scale imaging

Arrays of nanoscale light-emitting diodes (LEDs) offer an energy-efficient means for achieving microscopic imaging with resolution below the diffraction limit. Microscale LEDs are already poised to have a transformative impact on display technology, but it remains challenging to construct arrays of individually controllable LEDs at the nanometer scale. Daria Bezshlyakh of the Technische Universität Braunschweig in Germany and colleagues have developed a fabrication approach that enabled them to manufacture a compact super-resolution imaging system based on such arrays. Their device employs gallium nitride nano-LEDs that generate precisely structured patterns of illumination, which make it possible to image specimens at spatial resolutions smaller than the wavelength of light. Based on the performance and stability of this initial prototype, the authors conclude that similar devices could offer a promising platform for future molecular imaging applications.

## Introduction

GaN LEDs have revolutionized lighting technology because of their outstanding efficiency and versatility. Currently, optoelectronics based on GaN are developing even beyond solid-state lighting and inspire a family of new devices linked to microLEDs, enabling a variety of interesting applications, with display technologies being the main driver. The ability to produce even smaller LEDs in the micron range and below is a particular capability related to the physics of nonradiative carrier recombination at GaN surfaces. Due to the relatively slow GaN surface recombination, such GaN microLEDs have a high efficiency even at very small dimensions, upon which the surfaces usually play a dominant role in carrier recombination. This is in contrast to conventional InGaAlP LEDs, where the efficiency is substantially decreases for dimensions smaller than 20 µm^1,2^.

The potential impact of microLED technology on our society is substantial. For example, it is expected to become a core technology for TV or smartphone displays, where dimensions between 10 and 100 µm are used. Such microLEDs are placed individually in a mechanical pick-and-place approach on a large screen with a very low fill factor. Even though reliable assembly technologies are still a challenge, it is safe to expect this disruptive technology to replace OLED and LCD display technologies in the future. High-density and fully integrated microLEDs can be used for microdisplays with sizes of 1 cm^2^ and below in augmented reality applications, where high efficiency, high brightness, robustness, and degree of integration are key requirements. Such microdisplays can be made of microLEDs with dimensions below 10 µm and fully integrated on a single chip with a high fill factor^3–5^.

To the best of our knowledge, there are no reports to date on a functional array of nanosized LEDs with individual control of all array elements and a high fill factor. Demonstrated nanoLED fabrication methods, such as dry etching of LED nanopillars^6^ and selective growth of nanostructures^7^, has been used to successfully process LEDs as small as 100 nm. However, both methods are not suitable for an arrangement of structures in dense arrays, which would result in very complex addressing schemes. On the other hand, reported LED sizes in individually controllable arrays that are targeted for microdisplay technologies, start from 5 µm, as a greater size reduction would not be suitable for integration with ASICs, which are used for active LED driving^8,9^. Here, we report on a strategy to go beyond existing approaches by developing even smaller LEDs with dimensions comparable or smaller than the wavelength of visible light, targeting dimensions of 500 nm and below. Having such small dimensions, these arrays are obviously no longer meant for microdisplays, since the pixel-to-pixel distance is smaller than the typical dimensions given by Abbe’s diffraction limit, and single pixels can no longer be distinguished with optical systems. However, the capability of switching single nanoLEDs separately in an array at such small dimensions allows the light to be controlled at the nanoscale with a spatial precision below the wavelength of light and possibly even below the diffraction limit.

Having nanoscale light engines available opens up a broad landscape of applications, targeting the area of super-resolution. A platform of several individually switchable nanoLEDs with a nanoscale pitch allows the creation of light patterns with unprecedented spatial resolution. Applications of such chip-based structured illumination systems extend from the emission of controlled light patterns for twisted light to spatially resolved illumination in optogenetics experiments. Structured illumination platforms and their applications have already been described in the literature but are generally based on macroscopic devices, like optical fiber bundles or laser systems with complex control functions and a relatively low spatial resolution^10,11^. In this work, we demonstrate the development of nanoLED arrays that, as a single-chip technology, can be highly integrated and miniaturized and can increase the degree of flexibility, robustness, scalability, and availability of structured illumination platforms.

**Fig. 1:**
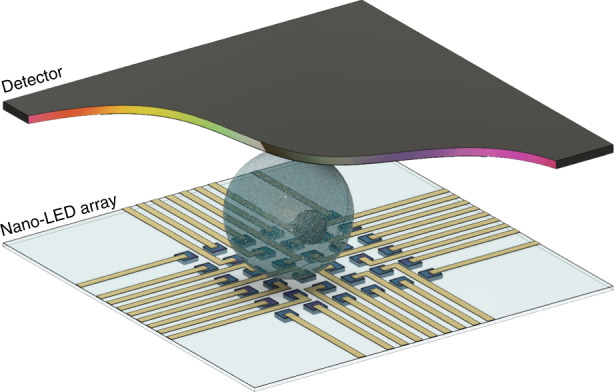
Sketch of a nano-LED array-based structured illumination microscope: a specimen is placed in close contact with an array of separately switchable point-like light sources (nano-LEDs) and a detector. Structured illumination or pixel-to-pixel scanning allows the acquisition ofstructural information for a small area of the sample at a time.

As a concrete application example, we are targeting a whole new approach to optical microscopy using structured illumination at the nanoscale. In conventional optical microscopy, the sample is homogenously illuminated and emits stray light, which is spatially resolved by a camera sensor after passing through a magnifying optical system. In this case, the illumination is homogeneous, whereas the spatial resolution is introduced by the detector system that consists of lenses and a spatially resolving image detector. In our approach, this principle is reversed: a nanostructured light source with homogenous distribution of individually addressable emitters enables illumination with a high spatial resolution. As a consequence, there is no need for the detection system to introduce spatial resolution, since this is already provided by the illumination^12^. This means that complex lens systems, which are typical for microscopy systems, are no longer needed. The overall system is made from only two semiconductor chips: a nanoLED structured illumination device and an integrating optical detector, which are the key components of a chip-based microscope. Its spatial resolution scales with the pixel pitch of the structured illumination platform, as is shown later. The overall setup is shown schematically in Fig. 1. In this configuration, the recorded intensity at the sensor directly depends on the transmissivity of the light path from the corresponding pixel to the sensor, which is related to the optical characteristics of the sample. If the specimen and the light sources are in close contact, the optical resolution is mainly defined by the pixel spacing of the array. Since the pixel-to-pixel distances presented here reach length scales below the diffraction limit, those nanostructured LED emitters are expected to pave the way towards a novel, compact super-resolution microscopy technology.

This paper focuses on the fabrication of nanoLED arrays with single pixel control as key elements for future chip-based super-resolution microscopes, followed by a thorough analysis of the optical and electrical properties. Details on the performance as a chip microscope can be found in related publications^13,14^.

## Results and discussion

### Process description

In comparison to that for single nanoLEDs^15^ and arrays with rather large pitches^6^, nanoLED arrays with pitches in the submicrometer regime and direct addressing of every single nanosized pixel bring along a whole new level of complexity to the fabrication process. Here, we describe the fabrication process of nanoLED test architectures using two different array configurations: (1) a 6 × 6 array with 400 nm nanoLEDs and (2) a 2 × 32 array with 200 nm nanoLEDs. The spacing between the neighboring pixels is kept equal to the LED size. The main challenge for fabricating such lines is providing every single LED of the array an individual contact line and avoiding short circuits and pixel crosstalk at dimensions of only 200 nm on GaN LED wafers, which are known to be characterized by high defect densities, a relatively high roughness and a pronounced nonisotropic chemical behavior^16^. This is in strong contrast to silicon wafers with their ideal, defect-free lattice structure, and perfectly known etching behavior.

The definition of addressable pixels in an array can be realized in two ways: by separating the LEDs physically or by making use of the low conductivity of p-GaN. In the latter case, the current spreading under a metal contact is so small that the nanoLED size is mostly defined by the size of the metal contact, without the need to separate single nanoLEDs by etching. In contrast, the first approach would require deep etching and planarization of the high-aspect ratio structures, since the lateral dimensions target 200 nm and the overall thickness of the GaN thin film structure on top of the isolating sapphire substrate is approximately 5 µm. Under such conditions, controlled etching and refilling on a submicrometer scale is difficult to achieve^17^. Therefore, a metal-oxide-GaN (MOGaN) process is developed for fabrication of nanoLED arrays. The MOGaN process takes advantage of the limited p-type conductivity, which is a general property of p-GaN layers. In addition, p-GaN layers are always the top layer of the LED structure. As a consequence, current spreading under the p-contact is quite limited and depends on the p-conductivity as well as the layer thickness, which restricts the light emitting area without the necessity of isolating the nanoLEDs from each other. More details about the light emitting area of a MOGaN nanoLED is discussed later in the analysis section.

**Fig. 2:**
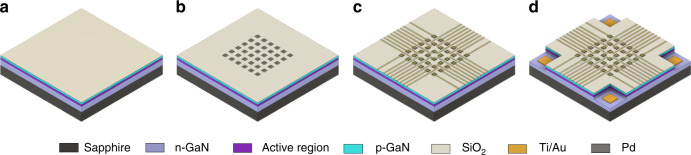
Phases of the MOGaN nanoLED array fabrication process: **a** deposition of SiO_2_ insulation layer on the p-GaN LED surface, **b** dry etching of the SiO_2_ layer for pixel opening and pixel filling with a thin Pd film, **c** fabrication of a Ti/Au p-contact line pattern, and **d** deep etching and metallization of the n-contacts.

The whole fabrication process combines photolithography and electronbeam lithography, dry and wet etching, and dielectric and metal evaporation. As shown in Fig. 2, it can mainly be split into four steps: (a) insulation, (b) pixel opening, (c) wiring, and (d) etching down to the n-buffer as common n-contact.

A key component of the MOGaN approach is a structured insulating SiO_2_ layer on top of the p-GaN layer. Metal contact lines can be designed on top of the SiO_2_, physically contacting the p-GaN only where openings in SiO_2_ have been fabricated by SiO_2_ etching. This produces contact the p-GaN in these limited small areas only, which serve as the LED area in the final device.

As an insulating layer, 75 nm of SiO_2_ is deposited onto the p-GaN surface of the LED wafer via plasma-enhanced chemical vapor deposition (PECVD). The patterning of SiO_2_ involves electron beam lithography (EBL), inductively coupled plasma reactive ion etching (ICP-RIE) of holes into SiO_2_ and metal deposition to fill the holes in the SiO_2_. The combination of EBL and etching of such small dimensions is critical and was thoroughly optimized. Dry etching was carried out at room temperature in an Ar/CHF_3_ atmosphere^18^. This results in a 1:2 etch ratio between the SiO_2_ and PMMA EBL resist, which means that at resist with a thickness of at least 200 nm required for the process. Next, the openings are filled with palladium (Pd) via electronbeam physical vapor deposition, followed by a lift-off process. Previous studies demonstrated that the Pd/Au stack shows the lowest contact resistance to p-GaN (4.3 × 10^−4^ Ω cm^2^) compared to that of other Pd-based and Pt-based metal combinations^19,20^.

**Fig. 3:**
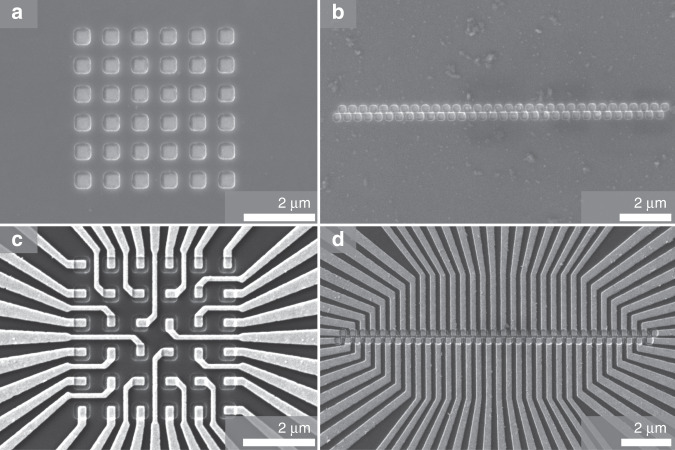
SEM images of etched pixels (top) and final structures (bottom) of the two geometries: **a**, **c** 6 × 6 array of 400 nm nanoLEDs and **b**, **d** 2 × 32 array of 200 nm nanoLEDs.

The resulting structures are too small to be resolved in an optical microscope, and hence, they have to be analyzed by scanning electron microscopy (SEM). The results are shown in Fig. 3 for both designs (6 × 6, 400 nm and 2 × 32, 200 nm) after opening of the holes in the SiO_2_ layer (a and b) and after deposition of the metal lines (c and d).

Once the geometry of the array is defined, a pattern of contact lines is produced to directly address each LED. Since the geometry of the array implies a challengingly small pitch (distance = pixel dimension), the positioning of the contact lines has to be done with the highest precision so that they fit in between the pixels without any cross-talk or electrical shorts. Pattern misplacement or wire enlargement can cause interconnections between several LEDs. Therefore, an overlay accuracy of below 50 nm and a thorough correction of proximity effects are required. The line pattern is metalized with a 5/100 nm Ti/Au stack via metal evaporation and lift-off. Deep-etching of GaN for n-contact formation (one common contact for all pixels) finalizes the fabrication process. In Fig. 3c, d, SEM images of the produced nanoLED arrays are depicted. After the optimization of all processing steps, good reproducibility is achieved.

The LED emission is highly divergent and generated in the active region, which is located 100 nm below the top surface (100 nm p-GaN layer), so the preservation of a high spatial resolution requires light outcoupling through the top surface of the LED. Therefore, the p-contact pad must be transparent or semitransparent for the wavelength of the emitted light. Optical transparency of the Pd/Ti/Au stack in the proposed design is achieved by reducing its thickness to 20/5/100 nm. Efficient top light outcoupling is demonstrated in the characterization section.

**Fig. 4:**
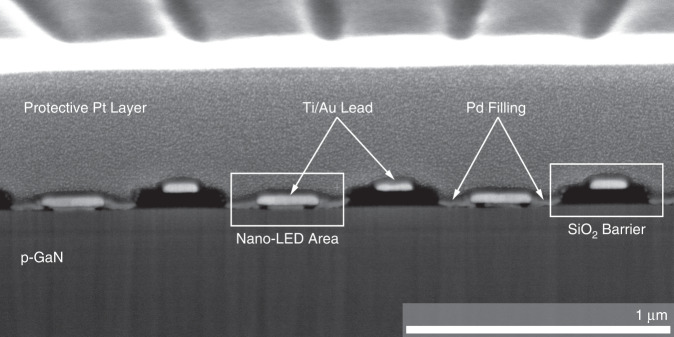
SEM image of a cross-section of 400 nm nanoLEDs. The cross-section was created with a focused ion beam. NanoLEDs are formed in the contact area between the Ti/Au lead and p-GaN. The image demonstrates a clear separation of the nanoLEDs by the SiO_2_ barrier. A thick Pt layer is deposited on the sample surface to prevent the ion-beam induced damage.

In addition to an exact lateral definition of all patterns during EBL, the interface quality between the p-GaN, metal contact, and metal leads also plays an important role during the fabrication process. Underetching of the SiO_2_ layer or low adhesion between the contact line and the semiconductor surface may lead to failure of the metal/semiconductor electrical contact. The analysis of this critical part of the device structure was carried out with a dual-beam microscope, which uses a focused (Ga) ion beam (FIB) and an electron beam for sample modification and observation. The FIB/SEM combination enables material removal with FIB at the highest positioning precision, accompanied by SEM imaging of the cross-section surface (Fig. 4). To prevent structural damage during FIB cutting, a thick Pt layer was deposited on the top surface of the array^21^. It can clearly be seen in the FIB cross-section that the SiO_2_ barrier separates the neighboring pixels well and prevents current leakage between the neighboring contact lines and p-GaN. The openings in the insulation layer are filled with a 20 nm Pd film and contacted with a Ti/Au lead. The FIB analysis demonstrates the high quality and good spatial definition of the nanoLEDs from our MOGaN process.

### Electro-optical characterization

To check the quality and the lateral extension integrity of the p–n junction, which is closely related to the light emitting area of the nanoLED, the electron beam induced current (EBIC) is measured under reverse bias conditions for single nanoLEDs^22^. When a reverse biased p–n-junction is exposed to an electron beam, charge carriers are generated and separated by the electrical field of the junction, which is enhanced due to the applied reverse bias voltage. Therefore, upon using an EBIC signal for spatial mapping, the extension and integrity of a p–n junction can be checked.

**Fig. 5:**
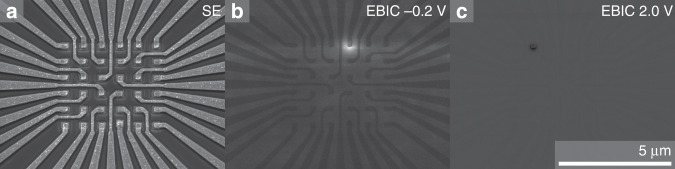
EBIC characterization of 400 nm nanoLEDs. **a** SEM and **b** EBIC images of 400 nm LEDs under reverse bias of −0.2 V, and **c** forward bias of +2 V.

Figure 5b showsthat the strongest EBIC signal occurs only below the contact area of the nanoLED under reverse bias, indicating that no voltage cross-talk between neighboring lines exists. In addition, the area of the nanoLED under forward bias fits well to the geometrical area of the hole in SiO_2_, which indicates that carrier diffusion is small.

**Fig. 6:**
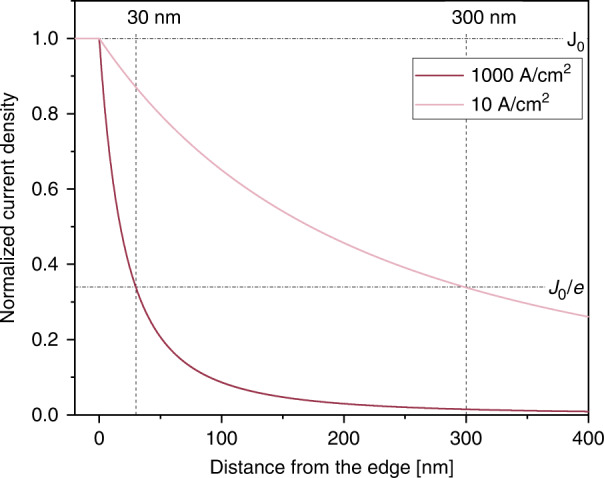
Dependence of the current density in the current spreading layer on the distance from the metal contact pad edge for two current density values (in the inset: *J*_0_ = 1000 and 10 A/cm²). Dashed lines mark the current spreading length, which is the distance where the current density reaches the *J*_0_/*e* value.

To check the consistency of our results, we also estimate the emission area of a nanoLED by calculating the current spreading in the p-GaN with respect to the metal contact pad size. For the LED configuration in this work, when the LEDs have a common n-contact and each pixel has an individual p-contact, light is emitted not only under the p-pad but also in a limited area around it. This area is defined by carrier diffusion, and the carrier concentration decays exponentially with increasing distance from the contact. Therefore, the current spreading in the p-GaN layer is an important way to measure the pixel size^23^. The value of lateral current spreading in p-GaN is expected to depend on the p-GaN layer thickness and the electrical resistivity of the p-GaN layer [Eq. 1].

1 $$L_{\mathrm{s}} = \sqrt {\frac{{tn_{{\mathrm{ideal}}}kT}}{{\rho J_0e}}},$$

where *L*_s_ is the current spreading length, *t* is the thickness of the p-layer, *n*_ideal_ is the ideality factor of the p–n junction, *ρ* is the resistivity of the p-GaN, *J*_0_ depicts the current density in the contact area, and *e* is the elementary charge. In Fig. 6, the current density in the current spreading layer is plotted as a function of the distance from the edge of the metal contact. According to the theory of current spreading^24^, the current spreading length, defined as the distance between the metal edge position and the point, where the current density reaches 1/*e* of the original current density *J*_0_, depends on the current density and decreases as the current density increases. According to Eq. 1 and assuming typical values for our GaN LEDs (*ρ* = 0.45 Ωm, *t* = 100 nm, and *n*_ideal_ = 1.3), *L*_s_ is as low as 30 nm for a 1000 A/cm^2^ original current density and increases to 300 nm for a 10 A/cm^2^ original current density. In conclusion, the current spreading increases the light emitting area by approximately 20% relative to the original area of the metal contact under targeted operation conditions of 1000 A/cm².

**Fig. 7:**
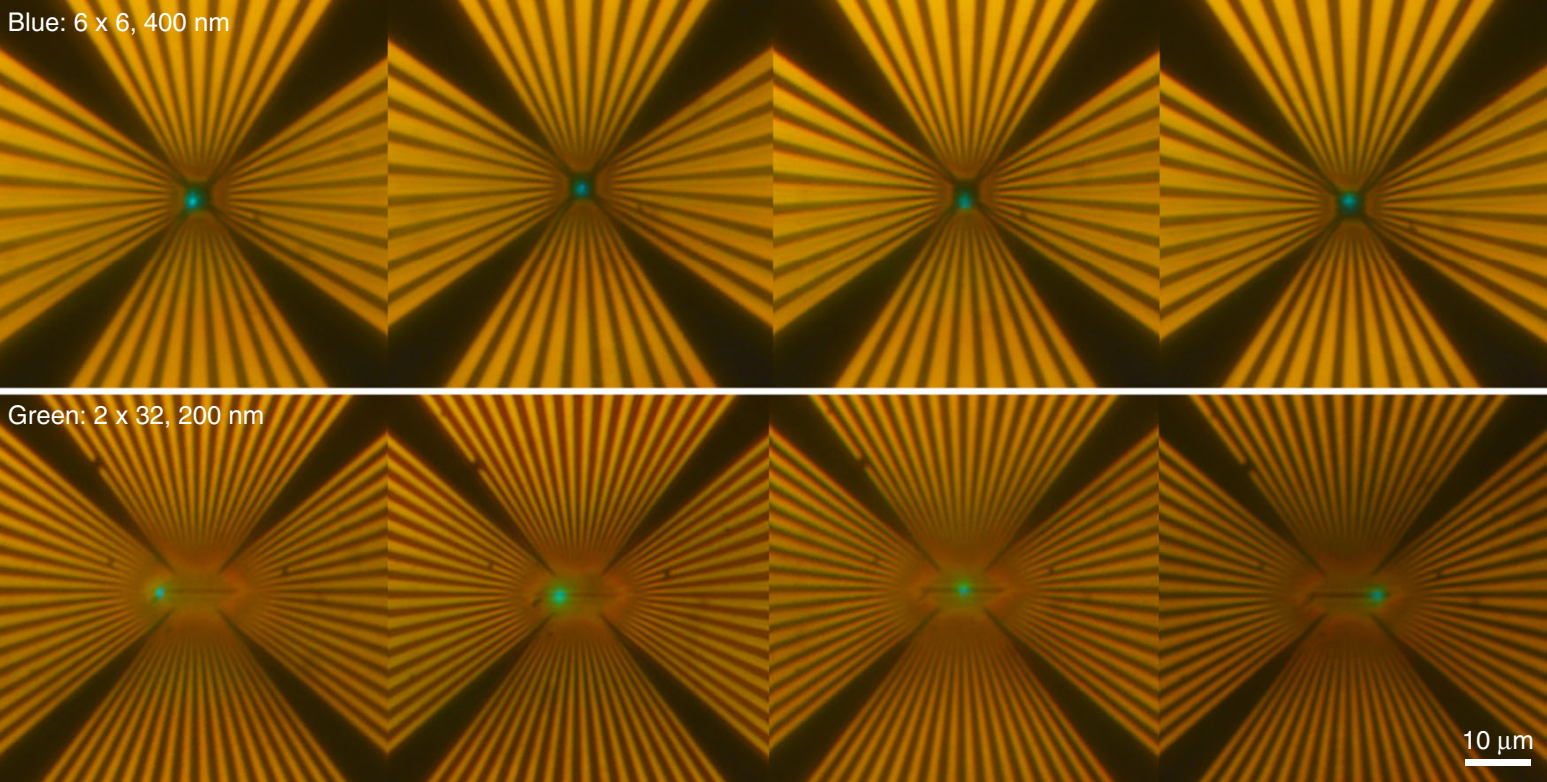
Optical images of the nano-LED arrays of both geometries: 6 × 6 array with 400 nm LEDs, blue-emitting (top); and 2 × 32 array with 200 nm LEDs, green-emitting (bottom). The images are taken with 50× magnification. A shift of the emissive region can be detected. However, the LEDs are too small for detailed investigation under an optical microscope.

The performance and physical properties of the nanoLED array are studied by analyzing the current–voltage (I–V) characteristics. Since the dimensions of the produced LEDs are below the spatial resolution limit of a standard optical microscope (Fig. 7), electro-optical characterization isin a scanning electron microscope. The contacts to the p-pads and n-pads are realized by two tungsten nanoneedles mounted on micromanipulators. The scanning electron microscope with the nanoneedle setup is equipped with a cathodoluminescence system so that the light emitted by the LEDs is collected by a parabolic mirror and directed to an optical detection system utilizing a monochromator and a CCD camera. In this way, the emission spectra of single nanoLEDs can be measured in electroluminescence (EL) mode as a function of the current.

**Fig. 8:**
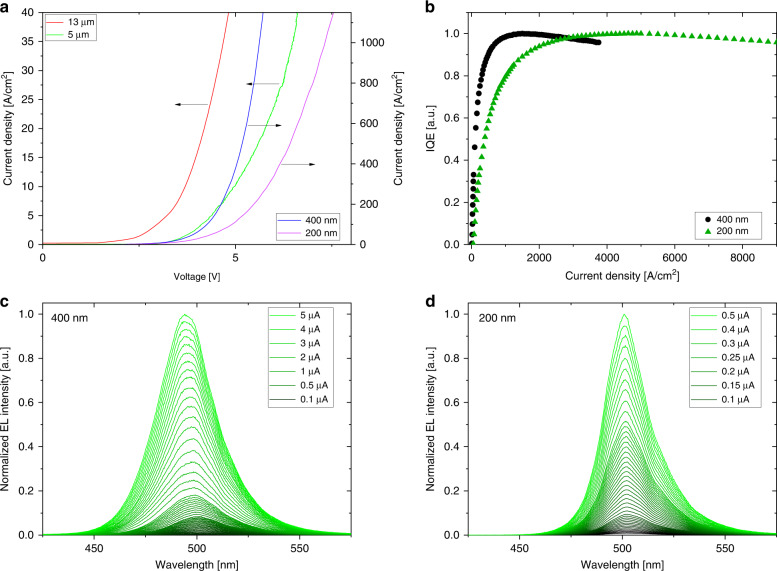
IVL characteristics of fabricated nanoLEDs. **a** IV curves of nanoLEDs compared to larger LEDs produced on similar wafersand, **b** normalized LED IQE vs. current density. Normalized EL emission spectra from **c** 400 nm and **d** 200 nm nanoLEDs for different driving currents.

A key question is whether the nanoLEDs are still intact, even at dimensions of only 200 nm and after various etching procedures during processing. Figure 8a shows the I–V characteristics fora set of GaN LEDs of different sizes. The LEDs compared in the graph areproduced with similar processes on the same InGaN LED wafers. Compared to that for state-of-the-art microLEDs, nanoLEDs turn on at higher voltages (4–6 V instead of 3 V). We attribute this to an increased contact resistance and, as shown, an influence of the contact line length, and a voltage drop due to the very small contact lines. The power consumption of the tested nanoLEDs lies in the microwatt range. It is 5 and 6 µW at a current density of 625 A/cm^2^, which corresponds to 1 and 0.25 µA driving current values for 400 nm and 200 nm LEDs, respectively.

Figure 8b shows the normalized IQE as a function of the current density. An efficiency droop can be observed at a current density of approximately 1 kA/cm^2^ for the 400 nm LEDs and 5 kA/cm^2^ for the 200 nm LEDs. Droop is known to be mainly caused by nonradiative Auger recombination^25^. The results indicate that the carrier densities in the active regions of the structures are not as high as those in LEDs in a large area with similar architecture. There, droop occurs above a current density of approximately 30 A/cm^2^. Multiple reasons are usually discussed for such behavior, such as substantial nonradiative channels leading to a very fast carrier decay or the appearance of parallel shunt resistances leading to an increase in the measured current. Analysis of the first and third quadrants of the I–V characteristic on a semilogarithmic scale shows no signs of parallel resistances^26,27^. For nanoLEDs, a “perimeter-adjusted” ABC model has recently been discussed^28^, where the maximum in a droop curve has been theoretically explained to occur at current densities of approximately 10^4^ A/cm² and higher for nanoLEDs with diameters of 100 nm. For the simulation, a pronounced nonradiative carrier decay has been assumed to occur at the sidewalls of the nanoLEDs. In our case, however, the nanoLEDs are not etched, so such sidewalls do not occur. In addition, by estimating the IQE of nanoLEDs using the ABC Model^29^, we achieve values between 5 and 30% for nanoLEDs that have a diameter below 1 μm compared to efficiencies of approximately 70% for macroscopic LEDs of the same epitaxial structure. Overall, the shift in the droop to current density values of kA/cm^2^, derived from purely geometric considerations, can presently not be explained conclusively. Additional work must be done to understand the droop behavior of our nanoLEDs.

In Fig. 8c, d, the emission spectra of a 400 nm and a 200 nm LED are shown. For both sizes, light emission can be observed at currents as low as 10 nA. With increasing current, a blue shift in the emission is observed. This is attributed to band filling effects and screening of the quantum confined stark effect, which occurs with standard-sized c-plane LEDs^30^.

## Conclusions

In this paper, we report on the fabrication and characterization of GaN nanoLED arrays as compact and flexible light engines for the generation of structured light patterns with submicron resolution. Advanced material processing techniques in combination with the advantageous properties of GaN allow the fabrication of arrays of light emitters, which are smaller than the wavelength of the light they emit and possess individual control of each single nanosized pixel. High structural quality and stable operation of nanoLEDs are demonstrated. The nanoLEDs are analyzed by multiple characterization methods with high spatial resolution, including FIB, EBIC, and nanoEL, and demonstrate a reasonable quality despite the very small dimensions. It has been demonstrated that GaN as a base material for future nanoLED technology has substantial advantages: slow surface recombination, low current spreading in p-GaN, the possibility of tuning the emission wavelength in the blue-green range, and the mature background in processing technology developed over the past years. This breakthrough has the potential to enable many novel applications in the future.

## Materials and methods

### Growth of GaN specimens by metal-organic vapor phase epitaxy (MOVPE)

The investigated LED specimens are grown by metal-organic vapor phase epitaxy on 430 µm thick 4” c-plane sapphire substrates using standard precursors, namely, trimethylgallium (TMGa), triethylgallium (TEGa), trimethylindium (TMIn), trimethylaluminum (TMAl), monosilane (SiH_4_), magnesocene (Cp_2_Mg), and ammonia (NH_3_), as well as hydrogen and nitrogen as carrier gases in an Aixtron AIX2600HT G3 24 × 2” planetary reactor.

The sapphire wafers are thermally cleaned within the MOVPE reactor in hydrogen at up to 1100 °C prior to a nitridation step and the deposition of a low-temperature GaN nucleation layer. Following a recrystallization and coalescence step, the deposition of a 4.4 µm thick n-GaN stack with silicon concentrations varying between 1 × 10^18^ and 3 × 10^19^ cm^−3^ occurs at a temperature of 1050 °C, reactor pressures between 125 and 290 mbar and a V/III (NH_3_/TMGa) ratio of approximately 1000 using hydrogen as the carrier gas. The active region is grown at a low temperature using TEGa and TMIn as group III precursors, a high V/III ratio of 22600 and a reactor pressure of 200 mbar. The MQW stack consists of a 10.5–11.0 nm quantum barrier grown at 795 °C and a 2.5–3.0 nm quantum well deposited at 740 °C with a TMIn/(TMIn+TEGa) inlet ratio of 0.45, both using nitrogen as the carrier gas. For the deposition of the 23 nm thick magnesium-doped Al_0.12_Ga_0.88_N electron blocking layer at 950 °C, the carrier gas is switched back to hydrogen. The LED structure is concluded by two 54 and 46 nm thick p-GaN layers with magnesium concentrations of 5 × 10^19^ cm^−3^ and 2 × 10^20^ cm^−3^, respectively. To activate the magnesium acceptors, in situ annealing is carried out in the growth reactor under a nitrogen atmosphere at 600 °C for 20 min.

### Electron beam lithography

Fabrication of the demonstrated nanoLEDs includes three EBL steps on an EBPG 5200 (Raith B.V.) at 100 keV and using PMMA lift-off processes.

In the first step, marker structures are created that serve as references for the next EBL steps. For this purpose, the wafer is coated with 120 nm PMMA (Allresist P 672.03) and a 30 nm chrome layer. This chrome mirror is necessary to be able to carry out a height measurement during the EBL on the transparent wafer and to avoid charging of the substrate. After EBL exposure, the chrome mirror is first removed using chrome etching, and then the PMMA is developed at room temperature for 30 s in a mixture of MEK-MIBK and IPA developer (1% MEK, 24.7% MIBK, 74.2% IPA). The markers are then created from 5/30 nm Ti/Au via vapor deposition and lift-off processes.

In the next step, the LED pixel structures are produced using EBL. For this purpose, the wafer is coated with approximately 450 nm PMMA (Allresist P 672.06) and with a conductive protective layer (Allresist Electra AR-PC 5090.02). This conductive protective layer is necessary to prevent the charging effects of nonconductive wafers in the EBL. After exposure, the conductive protective layer is removed under flowing pure water, and the wafer is developed in the above-mentioned developer at room temperature for 90 s.

As a last step, the wiring of the LED pixels is created. For this purpose, the wafer is coated with 250 nm PMMA (Allresist P 672.045) and again with a conductive protective layer (Allresist Electra AR-PC 5090.02). After exposure of the wiring pattern in the EBL, the conductive protective layer is removed under flowing pure water, and the wafer is developed in the above-mentioned developer at room temperature for 60 s.

### Photolithography

Fabrication of the large structures is carried out via photolithography on the SUSS MJB4 manual mask aligner. The photoresist AZ5214E and corresponding developer AZ726 are purchased from Microchemicals GmbH.

### Dry etching

Dry etching of the SiO_2_ isolation layer for p-contact and GaN for n-contact is performed using the SENTECH SI 500–300 ICP plasma etching system. Selective etching of SiO_2_ with PMMA as an etching mask is carried out in a mixture of Ar (35 sccm) and C3HF4 (20 sccm) at −10 °C, 5 Pa reactor pressure, 500 W ICP power and 200 W HF power. The measured etch rates reach 15 and 30 nm/min for SiO_2_ and PMMA, respectively. The GaN etching recipe, in turn, requires Cr as an etching mask material. It is carried out in a 4 sccm SF6 and 50 sccm H2 gas mixture at room temperature, 0.1 Pa reactor pressure, 1192 W ICP power and 300 W HF power. The GaN etch rate reaches at 100 nm/min.

### Wet etching

KOH wet etching is used to decrease the roughness of the n-GaN surface after dry etching. The samples are immersed for 20 min in 1 M KOH solution at 80 °C. The process is stopped by rinsing the sample with DI-water.

### FIB

The nanoLED cross-section study is performed using a Thermofisher Helios 5 UX Dual Beam microscope. The cross-section is cut with a Ga ion energy of 30 keV and an ion current of 20 nA. Afterwards, the cross section is polished with 30 keV Ga ions using a beam current of 240 pA. The secondary electron images are obtained using a through lens detector (TLD) with an electron energy of 5 keV and an electron current of 100 pA.

### EBIC and electroluminescence

The produced nanoLEDs are electrically and optically characterized inside a Tescan Mira 3 GMH FE-SEM. The electrical contact to individual pixels is established using Kleindiek MM3A micromanipulators equipped with low current measurement kits and connected to a Keithley 2636 source and measuring unit. A Gatan Mono CL 4 cathodoluminescence system equipped with an Andor MCD camera is used for light collection. LabView and Python control of the electrical setup and synchronization to the CCD camera enable automated acquisition of IVL curves inside the SEM.
